# When Do Paediatric Patients with Familial Hypercholesterolemia Need Statin Therapy?^*^

**DOI:** 10.34763/devperiodmed.20172101.4350

**Published:** 2017-05-29

**Authors:** Matylda Hennig, Agnieszka Brandt, Joanna Bautembach-Minkowska, Dominik Świętoń, Agnieszka Mickiewicz, Magdalenia Chmara, Bartosz Wasąg, Ewa Kamińska, Anna Balcerska, Janusz Limon, Andrzej Rynkiewicz, Marcin Gruchała, Małgorzata Myśliwiec

**Affiliations:** 1 Department and Clinic of Pediatric Diabetology and Endocrinology, Medical University of Gdańsk Gdańsk Poland; 2 Department of Radiology, Medical University of Gdańsk Gdańsk Poland; 3 Department and Clinic of Cardiology, Medical University of Gdańsk Gdańsk Poland; 4 Department of Biology and Genetics, Medical University of Gdańsk Gdańsk Poland; 5 Department of Farmacology, Institute of Mother and Child, Warsaw Poland; 6 Department and Clinic of Pediatric, Hematology and Oncology, Medical University of Gdańsk Gdańsk Poland; 7 Department of Cardiology and Internal Medicine, University of Warmia and Masuria Olsztyn, Poland

**Keywords:** familial hypercholesterolemia, statins, ApoA/ApoB index, paediatrics, e-tracking, IMT, hipercholesterolemia rodzinna, statyny, indeks ApoB/ApoA, pediatria, etracking, IMT

## Abstract

**Introduction:**

Familial hypercholesterolemia (FH) is one of the most common autosomal dominant disorders. It is characterized by elevated LDL cholesterol levels occurring already by early childhood. Awareness of health risks in FH patients should incite health professionals to actively seek and treat children with lipid disorders to reduce their risk of myocardial infarction and stroke.

**Objective:**

The aim of the study was to evaluate the suitability of taking into account the following parameters: ApoB/ApoA index, IMT and e-tracking examination, when initiating statin therapy in FH patients.

**Materials and methods:**

The study included 57 male and female patients aged 9.57±3.2 years (ranging from 1 year to 17 years), diagnosed with familial hypercholesterolemia confirmed by molecular testing. All the participants had their lipid profile, ApoA and ApoB levels determined. Carotid intima-media thickness (IMT) was measured by carotid ultrasound and arterial stiffness was assessed by e-tracking. The dietary treatment efficacy was monitored in 40 patients and the 12-month combination treatment efficacy in 27 patients. The study was conducted prospectively and retrospectively. Statistical analysis was performed with the EPIINFO Ver. 7.1.1.14 statistical software package.

**Results:**

Patients with familial hypercholesterolemia had high mean levels of total cholesterol and LDL cholesterol (287±67 mg/dL and 213±73 mg/dL respectively). 34.37% of the study subjects had a markedly increased ApoB/ApoA index. On IMT or e-tracking examination all the subjects (100%) had vascular abnormalities. After 6 months of a low-cholesterol diet, the mean total and LDL cholesterol levels in the serum had been reduced by 7.2% and 6.2%, respectively. Statins in an average dose of 10.42±2.49 mg daily were prescribed to 36 patients. After one year of the statin therapy, the average serum total and LDL cholesterol levels were 203.5±34.8 mg/dL and 139.1±32.1 mg/dL, respectively, and were still above the target values. Moreover, side effects of the statin therapy were monitored. An increase in AST levels seen in the study group was not statistically significant. The mean creatine kinase level was within the range of normal. Moreover, in our study material we estimated the risk of cardiovascular events in relation to the ApoB/ApoA index. Higher cardiovascular risk was found in 34.37% participants.

**Conclusions:**

Increased risk of cardiovascular events based on ApoB/ApoA index and carotid e-tracking or IMT examination in paediatric patients with FH is an indication for statin therapy initiation.

## Streszczenie

**Wstęp:**

Hipercholesterolemia rodzinna jest jedną z najczęstszych chorób dziedziczonych w sposób autosomalny dominujący. Podwyższone stężenie frakcji LDL cholesterolu występuje już od wczesnego dzieciństwa. U pacjentów z hipercholesterolemią rodzinną świadomość istniejącego problemu powinna motywować do aktywnego poszukiwania i leczenia dzieci z zaburzeniami lipidowymi, celem zmniejszenia ryzyka wystąpienia w przyszłości zawału serca i/lub udaru.

**Cel:**

Celem pracy była ocena przydatności parametrów: współczynnik ApoB/ApoA, e-tracking oraz IMT, we wstępnej kwalifikacji pacjentów do leczenia farmakologicznego dzieci z hipercholesterolemią rodzinną.

**Materiał i metody:**

Do badania włączono grupę 57 pacjentów płci obojga w wieku od 1 roku do 17 lat, średnio 9,57±3,2 lat z rozpoznaniem hipercholesterolemii rodzinnej potwierdzonej badaniami molekularnymi. U badanych pacjentów oznaczono lipidogram, apolipoproteinę Bi A, a także grubość kompleksu błony wewnętrznej i środkowej oraz sztywność naczyń w badaniu e-tracking wykonując USG tętnic szyjnych. Badania były przeprowadzone zarówno retrospektywnie jak i prospektywnie. Obserwowano 40 pacjentów stosujących leczenie dietetyczne, a następnie 27 pacjentów leczonych dietą oraz statynami. Analizę statystyczną przeprowadzono wykorzystując komputerowy pakiet programów statystycznych EPIINFO Ver. 7.1.1.14.

**Wyniki:**

Pacjenci z hipercholesterolemią rodzinną charakteryzowali się wysokim średnim stężeniem cholesterolu całkowitego 287±67 mg/dl oraz cholesterolu LDL 213±73mg/dl. U 34,37% badanych stwierdzono podwyższony indeks Apob/Apoa. Nieprawidłowości w układzie naczyniowym w badaniu IMT bądź e-tracking wykazano u 100% badanych. Po stosowaniu diety niskocholesterolowej przez 6 miesięcy średnia wartość cholesterolu całkowitego w surowicy obniżyła się o 7,2%, cholesterolu LDL obniżyła się o 6,2%. Statyny otrzymało 36 pacjentów w dawce średnio 10,42±2,49mg/dobę. Po roku leczenia farmakologicznego średnie wartości stężeń w surowicy cholesterolu całkowitego wynosiły 203,5±34,8mg/dl oraz cholesterolu LDL 139,1±32,1mg/dl i były nadal powyżej normy. Ocenie poddano także skutki niepożądane leczenia statynami. Wzrost stężenia AST w badanej grupie nie był znamienny statystycznie. Średnia wartość kinazy kreatyninowej mieściła się w normie. W materiale własnym przeprowadzono także prognozę ryzyka wystąpienia zaburzeń kardiologicznych w odniesieniu do stężenia wskaźnika ApoB/ApoA. U 34,37% badanych stwierdzono zwiększone ryzyko wystąpienia powikłań sercowo-naczyniowych.

**Wnioski:**

Kwalifikacja pacjenta pediatrycznego z hipercholesterolemią rodzinną do grupy ryzyka powikłań sercowo naczyniowych w oparciu o wartość wskaźnika ApoB/ApoA oraz badanie e-tracking lub IMT tętnic szyjnych jest wskazaniem do podjęcia leczenia statynami.

## Introduction

Familial hypercholesterolemia (FH) is one of the most common and best studied monogenic diseases. It accelerates the progression of atherosclerosis and increases the incidence of cardiovascular events [1, 2]. Because of the inheritance mode (autosomal dominant) we distinguish two forms of the disease: heterozygous (HeFH) and homozygous (HoFH). Homozygous FH in the Caucasian population is seen in one per million live births, while the heterozygous form – on average in one per 300 to 500 live births [3].

The disease is a major health problem, because it is an initially asymptomatic disorder that finally leads to severe atherosclerosis and cardiovascular events. Cardiovascular mortality in these patients as early as at the age of 20 to 39 years is 100 times as high as in the general population [4].

Therefore, the identification of patients with FH in the general population and the development of precise treatment approaches at the early stage of the disease is of key importance.

## Objective

The aim of the study was to evaluate the suitability of parameters: ApoB/ApoA index, IMT and e-tracking in initiating statin therapy in FH patients.

## Materials and methods

The study sample included 57 children aged 9.57±3.26 years, with a molecularly confirmed diagnosis of familial hypercholesterolemia. At the time of presenting at the Lipid Clinic the youngest patient was 13 months old and the oldest one 17 years and 2 months.

The patients were treated with a low-cholesterol diet alone or in combination with a statin. The statins used were atorvastatin in the dose of 5 to 10 mg daily.

The study was conducted prospectively and retrospectively. All the patients had their total cholesterol, HDL cholesterol and triglyceride levels measured by standard analytical methods in the Central Laboratory of the University Medical Centre in Gdańsk. LDL cholesterol levels were calculated from the Friedewald equation, provided that the triglycerides levels did not exceed 350 mg/dL. Patients with confirmed lipid disorders underwent further diagnostic procedures including apolipoprotein (Apo) A and ApoB measurements and calculation of the ApoB/ApoA ratio, which is thought to reflect the risk of cardiovascular complications.

Data on diagnostic methods and therapy were gathered retrospectively on the basis of medical history and documentation analysis. The data included aspartate transaminase (AST), alanine transaminase (ALT) and creatine kinase (CK) levels, which were measured in smaller groups of patients (32, 29 and 17 subjects, respectively). Data were collected on liver enzymes and creatine kinase levels before and 6 weeks after statin therapy initiation. None of the patients reported any adverse events during the treatment period.

As part of treatment monitoring, lipid levels were checked again after 6 months of dietary treatment and after one year of drug therapy. The dietary treatment e' cacy was monitored in 40 patients and the 12-month combination treatment efficacy – in 27 patients (15 patients started combination treatment at the beginning and 12 patients after dietary treatment). 2 patients resigned from therapy.

### Imaging parameters

Before commencing the therapy, we measured the Intima-Media Thickness by carotid ultrasound and performed e-tracking, which enables early and noninvasive diagnosis of atherosclerotic lesions by evaluating vessel stiffness. Each patient was examined after a 5-minute rest, lying in a horizontal position, with their head tilted slightly away from the examined side. Examinations were performed with the Aloka Alpha 6 ultrasound machine, with the linear 8MHz transducer. Focal distances were rigid and enhancement chosen so as to minimize the number of artefacts in the vessel lumen. The transducer was placed perpendicularly to the common carotid artery. The measurements were taken 2 cm from the carotid bulb in both the left and the right carotid and then an average was calculated from readings of the two measurements. Blood pressure was measured on the brachial artery, using the automatic blood pressure monitor Omron M2. The cuff width was selected according to the participant’s arm circumference.

Afterwards the average IMT results were compared to the normative values elaborated by C. Jourdan et al. [5].

The examination was conducted in 52 patients at the start of treatment.

E-tracking evaluated the β-index, which is the ratio of the natural logarithm of BP changes to the vessel diameter changes. The index rises when vessels become more rigid.

The results obtained for e-tracking parameters were compared against reference values established by M.Calabro et al. [6]

This diagnostic test was performed in 40 patients at the beginning of treatment.

Imaging diagnostic tests were conducted at the Radiology Unit of the University Clinical Centre in Gdańsk, by a single ultrasound specialist.

The study was approved by the independent bioethics committee for scientific research at the Medical University in Gdańsk, No NKBBN 35/2013

The research was financed by the National Science Centre; decision No 04/0136/09/156

### Statistical analysis

The findings of the study were analysed by statistical methods to verify our hypothesis. The hypothesis on the equality of means from all samples was verified by ANOVA or by the nonparametric Kruskal-Wallis sum-rank test (for anomalous groups or groups of small number of cases); the homogeneity of variance was estimated by Bartlett’s test.

The hypothesis that the parameter means in dependent samples (before and after the treatment) are equal was verified by Student’s t-test for paired samples for groups with normal distribution, and by Wilcoxon signed-rank test when populations cannot be assumed to be normally distributed; normality was tested by the Shapiro-Wilk test.

For specified pairs of parameters the correlation analysis was done by calculating the Pearson correlation coefficient r, where P ‹ 0.05 was considered statistically significant. Statistical analysis was performed using a statistical program package EPIINFO Ver. 7.1.1.14 (of 2^nd^ July 2013).

## Results

In the study sample (n=57) total and LDL cholesterol levels were elevated, while HDL cholesterol and triglyceride values remained within the normal limits when compared to the physiological norms. The statistical analysis showed no significant correlation between the patients’ age and total cholesterol, or any of its fraction levels (Tab. I).

**Table I:** Correlation between the patients’ age and total cholesterol or any of its fraction levels (n=57). Tabela I. Korel acja wieku pacjenta ze stężeniem cholesterol oraz jego frakcji (n=57).

Group *Grupa*	age *Wiek*	N=57	Total cholesterol [TC]/*Cholesterol całkowity*	LDL	HDL	TG
**1**	<6 lat	8	291.0	238.0	42.5	855
**2**	6-10	22	262.5	189.5	48.0	75.0
**3**	10-15	19	267.0	208.0	56.0	86.0
**4**	>15	8	271.5	206.5	49.0	79.0

P	Group/*Grupa 1 vs 2 vs 3 vs 4*	Group/*Grupa 1 vs 2*	Group/*Grupa 1 vs 3*	Group/*Grupa 1 vs 4*	Group/*Grupa 2 vs 3*	Group/*Grupa 2 vs 4*	Group/*Grupa 3 vs 4*
TC	0.488	0.111	0.353	0.208	0.638	0.851	0.791
TG	0.611	0.189	0.791	0.401	0.440	0.851	0.457
LDL	0.559	0.159	0.339	0.345	0.565	0.760	0.853
HDL	0.119	0.336	0.0524	0.494	0.0439	0.981	0.288

Moreover, we estimated the risk of cardiovascular events in relation to the ApoB/ApoA index.

We measured ApoA and ApoB levels in 32 patients and calculated the ApoB/ApoA ratio. The lowest value was 0.44, the highest one − 1.48, and the average one 0.88±0.26. The average value was within the laboratory norm. In 11 patients, however, the value of this ratio exceeded the norm.

We found a significant positive correlation between the ApoB/ApoA ratio and total and LDL cholesterol levels (p=0,003), as well as a significant negative correlation between this ratio and the HDL cholesterol level (p=0,009).

Further analysis revealed a significant correlation between the ApoB/ApoA (n=17) ratio and Young’s modulus as well as with the e-tracking focal, one-point pulse wave velocity.

**Fig. 1:**
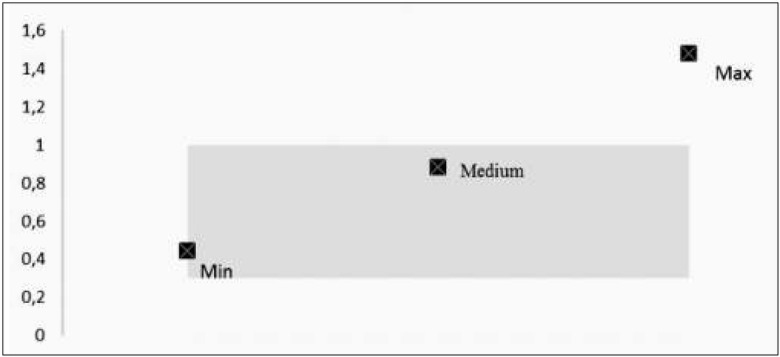
Minimum, maximum and average ApoB/ApoA index values respective to the norm (0.3-1) (n=32). Ryc. 1. Wartość średnia, minimalna i maksymalna wskaźnika ApoB/ApoA w odniesieniu do norm (0,3-1) (n=32).

The ApoB/ApoA ratio shows how advanced the atherosclerotic lesions are. In the studied group (n=32), the risk of cardiovascular complications was increased in 34.37% (11 participants) (Fig. 1).

### Ultrasound evaluation of atherosclerotic lesions

Ultrasound evaluation of the intima-media complex thickness (IMT) in the common carotid arteries was performed in 51 patients while e-tracking of the ß-stiffness of those vessels - in 40 participants.

**Fig. 2:**
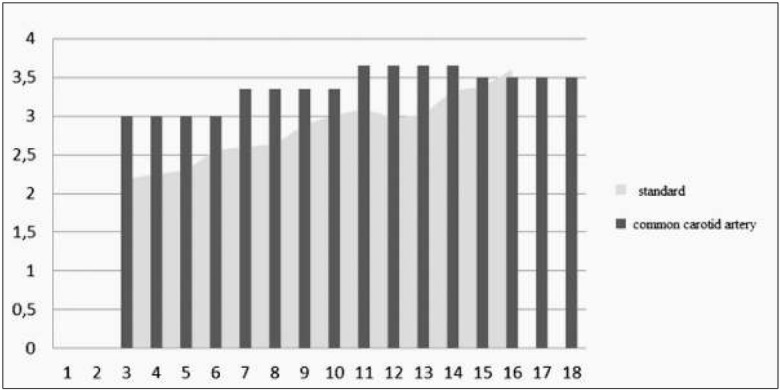
Analysis of the beta-stiffness index changes of the common carotid with reference to physiological norms and the patient’s age (n=40). Ryc. 2. Analiza zmiany wskaźnika sztywności naczyń ß w tętnicy szyjnej w odniesieniu do norm fizjologicznych, w odniesieniu do wieku pacjenta (n=40).

**Fig. 3:**
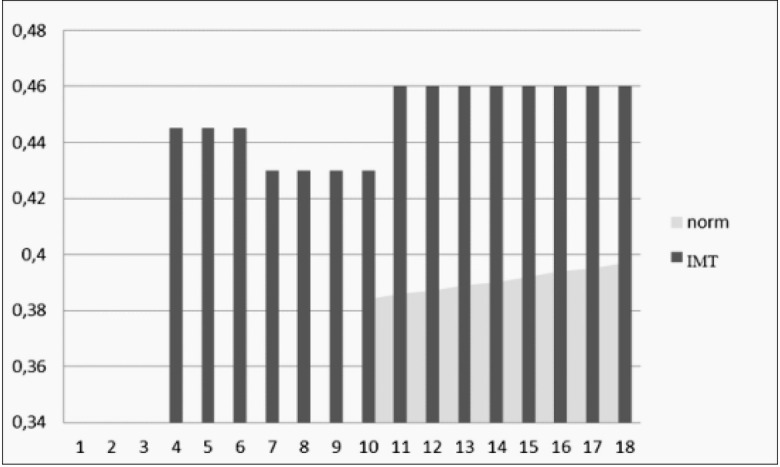
Analysis of the IMT changes of the common carotid with reference to physiological norms (n=51). Ryc. 3. Analiza zmiany grubości IMT w tętnicach szyjnych w odniesieniu do norm fizjologicznych (n=51).

Vascular abnormalities were reported in 100% of the participants as evaluated by the ß-stiffness index (in 40 patients) and the IMT (in 51 patients) (Fig. 2, Fig. 3).

We conducted a comparative analysis of total cholesterol and its fractions including LDL cholesterol, HDL cholesterol and triglycerides in 40 patients at baseline and again after 6 months of treatment with a low-cholesterol diet.

We observed a decrease in the mean serum total cholesterol level by 20 mg/dL (7.2%) and the LDL cholesterol level by 12.6 mg/dL (6.2%); the mean HDL cholesterol level decreased by 5.2 mg/dL (9.5%) and that of triglycerides – by 5.2 mg/dL (5.7%). The only difference that was statistically significant (p 0.00093) was that in total cholesterol levels before and after the low-cholesterol diet.

Although total cholesterol and LDL cholesterol levels were reduced, they remained above the upper limit of normal for this age group.

At the next stage we conducted a comparative analysis of particular lipid parameters in patients with diagnosed familial hypercholesterolemia after one year of dietary and statin treatment.

At 12 months of the therapy, changes in lipid parameters were reported in 27 patients. The average statin dose was 12.63±7.321 mg (range from 5 mg to 40 mg).

At the next stage of the study at 12 months of the statin therapy mean, the serum total cholesterol level decreased by 85.9 mg/dL (29.6%), the mean LDL cholesterol level − by 73.08 mg/dL (34.4%), the mean HDL-cholesterol level − by 7.65 mg/dL (13.13%), and the mean triglyceride level − by 28.6 mg/dL (28.2%). Statistically significant differences could be seen for total (p 0.0000006) and LDL (p 0.0000006) cholesterol concentrations, as well as for triglycerides (p 0.001453); the difference in HDL cholesterol concentrations was not statistically significant (p 0.455903). (Tab. II, Tab. III.)

**Table II:** Changes in lipid levels during their (6 month) dietary treatment n=40. Tabela II. Zmiany stężenia lipidów w trakcie leczenia dietą przez 6 miesięcy.

	** Total Cholesterol *Cholesterol całkowity mg/dl*	LDL mg/dl	HDL mg/dl	TG mg/dl
**Normal diet *Dieta ogólna***	276.9±54.1	204.5±62.1	54.6±27.6	91.5±37.7
**** Fat-modified diet *Dieta niskocholesterolowa***	256.5±49.1	191.9±46.5	49.4±9.9	86.3±39.4
p	0.00093	0.1	0.727	0.394

**Table III:** Changes in lipid levels during their (12 month) combination therapy with diet and statins n=27. Tabela III. Zmiany stężenia lipidów w trakcie leczenia dietą i statynami w ciągu 12 miesięcy.

	** Total Cholesterol *Cholesterol całkowity mg/dl*	LDL mg/dl	HDL mg/dl	TG mg/dl
Diet Dieta	289.4±59.84	212.2±71.16	58.22±31.9	101.2±64.3
12 month statin treatment *12 miesięcy leczenia statnami*	203.5±34.8	139.1±32.1	50.55±9.65	72.6±33.37
p	0.0000006	0.0000006	0.455903	0.001453

## Statin therapy and adverse effects

None of the patients reported any complaints during the combined diet and statin therapy. No delay or slowing down in sexual maturation was observed during the one year treatment period.

Moreover, we studied the adverse effects of statin therapy, liver function and muscle cell function, as monitored by the aspartate transaminase, alanine transaminase and creatine kinase levels during the six-week treatment.

Mean AST value increased from 22.6±5.5U/L to 23.4±7.3U/L. After 6 weeks of statin therapy we saw an increase above the upper limit of normal (54U/L) in one patient. The increase in AST values in the studied group was not statistically significant.

The mean ALT value increased from 17.2±8.5U/L to 19.4±10.0U/L. The increase in ALT values in the studied group was statistically significant.

Mean CK value decreased from 171.1±180.9U/L to 116.5±58.6U/L. The increase in CK values in the studied group was not statistically significant.

The following conclusions were drawn from the findings of the study.

## Discussion

Studies of FH patients who administered statins on top of their low cholesterol diet are relatively short-term, which makes discussion of the subject very difficult, especially when compared to the adult population.

Treatment decisions to start statin therapy were based on the following criteria: the patient’s age, total and LDL-cholesterol levels, ApoB/ApoA ratio and progression of atherosclerotic lesions as measured by carotid ultrasound e-tracking and IMT.

Initially low statin doses were prescribed with excellent treatment tolerance in the group of children from 10 to 18 years of age. After the 12-month treatment, serum total cholesterol levels decreased but not enough to fall within the normal range.

Depending on their doses, statins can reduce LDL-cholesterol levels by 10 to 60% [7, 8, 9].

Own observations also included the analysis of statin treatment results after another year of therapy. Total and LDL-cholesterol levels in the serum were still above the upper limit of normal. However, statins were much more efficient than a low-cholesterol diet alone.

The WHO report and expert analyses show that about 80% of cardiovascular diseases or strokes can be prevented with the elimination of major risk factors (WHO report: Gaining Health, 2006). [10] Therefore, it is of utmost importance in familial hypercholesterolemia to start lipid-lowering therapy as early as in childhood.

## Apob/Apoa ratio

Serum apolipoprotein measurements are done directly, while LDL-cholesterol concentration is calculated using the Friedewald formula.

The AIP (Atherogenic Index of Plasma) is indicative of the risk of cardiovascular complications. Judging from the bibliography, the ApoB/ApoA atherogenic index is also more closely correlated with the increase in the intima-media complex thickness than lipid values or other markers.

In studies by Wallidius et al. ApoB/ApoA measurements were of greater value for the estimation of the risk of stroke than LDL-cholesterol levels [11].

In our study 34.37% of the participants had an increased risk of cardiovascular complications. Starting pharmacotherapy in this group of patients is likely to reduce their risk of myocardial infarction or stroke in the future.

## Evaluation of the cardiovascular system in the study sample

The severity of atherosclerotic lesions can be monitored by IMT or e-tracking.

Jourdan et al. performed carotid ultrasound imaging in 250 healthy children and young adults between 10 and 19 years of age, calculated an average from the measurements of both the left and right carotid and established norms relative to age or height [5].

Our own observations have shown vascular abnormalities in 100% of the study participants, mostly in the form of increased IMT [5, 6].

E-tracking is also a non-invasive diagnostic tool that enables the evaluation of arterial stiffness, which can be indicative of atherosclerotic lesions long before the first clinical signs and symptoms occur. Calebro et al. performed this examination in a group of 130 healthy children, 3 to 16 years of age and based on those results norms for age were calculated [6].

Riggio et al. suggested that e-tracking may allow early detection of atherosclerotic lesions and that e-tracking coefficients show a positive correlation with total and LDL-cholesterol values [12].

In our study of familial hypercholesterolemia patients we reported an increased vessel stiffness coefficient versus the normal range.

## Evaluation of statin side effects

In general statins are well-tolerated. Possible adverse effects include indigestion, abdominal pains, flatulence and headache – seen in about 3-5% of the patients. There is also a slightly increased (0.5-2%) risk of liver damage. Therefore, it is very important to measure liver transaminases at baseline [12].

In the study by Wiegmann et al. or Nordestgaard et al. and Joyce no adverse effects, such as myopathy, increased transaminase levels or delayed growth or maturation were reported in children with statin use [7, 8, 9]. The results are consistent with our own materials. However, it must be emphasised that the data on statin use in children are short-term, so long-term consequences of such therapy cannot be foreseen [7, 8].

So far, the observations suggest that statin therapy in children carries a low risk of adverse effects and long-term consequences need to be clarified in further studies. Treatment with statins in children with familial hypercholesterolemia [14].

## Conclusions

1. Due to the high cost of molecular diagnosis, ApoB/ApoA ratio, carotid IMT or e-tracking in a pediatric patient with familial hypercholesterolemia can be useful, when taking the decision about indications for statin therapy.
2. The ApoB/ApoA ratio and e-tracking of intravascular lesions are valuable tools in evaluating the risk of cardiovascular complications.
3. After one year of treatment, it can be concluded that low-dose statin therapy in familial hypercholesterolemia patients is a safe but insufficiently effective method of lowering total and LDL cholesterol levels.

## References

[1] Austin MA, Hutter CM, Zimmern RL (2004). Familial hypercholesterolemia and coronary heart disease: a HuGE association review. American Journal of Epidemiology.

[2] Catapano AL, Reiner Z, De Backer G (2011). ESC/EAS Guidelines for the management of dyslipidemia of the European Society of Cardiology (ESC) and the European Atherosclerosis Society (EAS). Atherosclerosis.

[3] (1998). Familial Hypercholesterolemia. A report of a WHO consultation. WHO.

[4] Myśliwiec M, Walczak M, Małecka-Tendera E (2013). Stanowisko dotyczące postępowania w rodzinnej hipercholesterolemii u dzieci i młodzieży. Stanowisko Forum Ekspertów Lipidowych. Pediatria Polska.

[5] Jourdan C, Wühl E, Litwin M (2005). Normative values for intima-media thickness and distensibility of large arteries in healthy adolescents. Journal of Hypertension.

[6] Calabro MP, Carej S, Russo MS (2012). Normal paediatric values of arterial stiffness parameters measured by echo-tracking. The child, a Journal of Pediatrics.

[7] Wiegman A, Hutten BA, De Groot E (2004). E' cacy and safety of statin therapy in children with familial hypercholesterolemia. A randomized controlled trial. Jama.

[8] Joyce L Ross (2016). Statins in the Management of Pediatric. Journal of Pediatric Nursing.

[9] Nordestgaard BD, Chapman MJ, Humphires SE (2013). Familial hypercholestaerolemia is underdiagnosed and undertreated in the general population: guidance for clinicians to prevent coronary heart disease. European Heart Journal.

[10] Undas A (2010). Podolec P. Zaburzenia lipidowe − kogo i kiedy należy objąć badaniem przesiewowym. Podręcznik Polskiego Forum Pro#laktyki T.2. Kraków: Med Prakt.

[11] Walldius G, Aastveit AH, Junger I (2006). Stroke mortality and the apoB/apoA-I ratio: results of the AMORIS prospective study. Journal of Internal Medicine.

[12] Riggio S, Mandra' no G, Sardo MA (2010). Pulse wave velocity and augumentation index, but not intima-media thickness, are early indicators of vascular damage in hypercholesterolemic children. Eur J Clin Invest.

[13] Sobień B, Kopeć G, Podolec P. (2010). Statyny. Podręcznik Polskiego Forum Profilaktyki T.2. Kraków: Med Prakt.

[14] Kamińska E, Hennig M, Brandt A, Bautembach Minkowska J, Myśliwiec M (2016). Treatment with statins in children with familial hypercholesterolemia. Developmental Period Medicine.

