# Errattum To “When Do Paediatric Patients with Familial Hypercholesterolemia Need Statin Therapy?” [developmental Period Medicine. 2017;21(1):43-50. DOI: 10.34763/devperiodmed.20172101.4350]

**DOI:** 10.34763/jmotherandchild.20212501.erratum20172101

**Published:** 2021-10-11

**Authors:** Matylda Hennig, Agnieszka Brandt, Joanna Bautembach-Minkowska, Dominik Świętoń, Agnieszka Mickiewicz, Magdalenia Chmara, Bartosz Wasąg, Ewa Kamińska, Anna Balcerska, Janusz Limon, Andrzej Rynkiewicz, Marcin Gruchała, Małgorzata Myśliwiec

**Affiliations:** 1 Department and Clinic of Pediatric Diabetology and Endocrinology, Medical University of Gdańsk Gdańsk Poland; 2 Department of Radiology, Medical University of Gdańsk Gdańsk Poland; 3 Department and Clinic of Cardiology, Medical University of Gdańsk Gdańsk Poland; 4 Department of Biology and Genetics, Medical University of Gdańsk Gdańsk Poland; 5 Department of Farmacology, Institute of Mother and Child, Warsaw Gdańsk Poland; 6 Department and Clinic of Pediatric, Hematology and Oncology, Medical University of Gdańsk Gdańsk Poland; 7 Department of Cardiology and Internal Medicine, University of Warmia and Masuria Gdańsk Poland

In the article [Matylda Hennig, Agnieszka Brandt, Joanna Bautembach-Minkowska, Dominik Świętoń, Agnieszka Mickiewicz, Magdalenia Chmara, Bartosz Wasąg, Ewa Kamińska, Anna Balcerska, Janusz Limon, Andrzej Rynkiewicz, Marcin Gruchała, Małgorzata Myśliwiec. When do paediatric patients with familial hypercholesterolemia need statin therapy? Developmental Period Medicine. 2017;21(1):43-50. doi: 10.34763/devperiodmed.20172101.4350. PMID: 28551692.] there was an error regarding the financing of the work. Correct data provided again by the authors are published below:

The project was financed from the funds of the National Science Center granted on the basis of decision number DEC-2013/09/B/NZ5/02786.

Projekt został sfinansowany ze środków Narodowego Centrum Nauki przyznanych na podstawie decyzji numer DEC-2013/09/B/NZ5/02786.

